# Clinical and molecular evaluation of patients with ovarian cancer in the context of drug resistance to chemotherapy

**DOI:** 10.3389/fonc.2022.954008

**Published:** 2022-08-05

**Authors:** Marcin Opławski, Agata Średnicka, Ewa Niewiadomska, Dariusz Boroń, Piotr Januszyk, Beniamin Oskar Grabarek

**Affiliations:** ^1^ Department of Gynecology and Obstetrics with Gynecologic Oncology, Ludwik Rydygier Memorial Specialized Hospital, Kraków, Poland; ^2^ Department of Gynecology and Obstetrics, Faculty of Medicine and Health Sciences, Andrzej Frycz Modrzewski University in Kraków, Kraków, Poland; ^3^ Department of Epidemiology and Biostatistics, School of Health Sciences in Bytom, Medical University of Silesia, Katowice, Poland; ^4^ Department of Histology, Cytophysiology and Embryology, Faculty of Medicine, University of Technology, Academia of Silesia in Katowice, Zabrze, Poland; ^5^ Department of Gynecology and Obstetrics, Faculty of Medicine, University of Technology, Academia of Silesia in Katowice, Zabrze, Poland; ^6^ Department of Biochemistry, Faculty of Medicine, University of Technology, Academia of Silesia in Katowice, Zabrze, Poland; ^7^ GynCentrum, Laboratory of Molecular Biology and Virology, Katowice, Poland

**Keywords:** ovarian cancer, CA-125, HE4, mRNA, miRNA

## Abstract

The present study aimed to evaluate changes in the expression patterns at the gene and protein levels associated with drug resistance. The study group included 48 women who had a histopathologically confirmed diagnosis of stage I-IV ovarian cancer, they were divided into two subgroups (groups A and B). In group A, there were 36 patients in whom surgical treatment was supplemented with first-line chemotherapy according to current standards. Within this patient group, 5 had stage I (14%), 5 had stage II (14%), 25 had stage III (69%), and 1 had stage IV ovarian cancer (3%). Drug resistance was found after the third cycle of chemotherapy in 17 patients (71%) and after the sixth cycle in 7 patients (29%). Group B included 12 women with type I ovarian cancer, including 11 with stage I and 1 patient with stage IV ovarian cancer. The oncological treatment required only surgery. The control group (C) included 50 women in whom the uterus and adnexa were surgically removed for non-oncological reasons. Significantly higher levels of carcinoma antigen 125 CA-125 and human epididymis protein 4 HE4 were observed in group A and in menopausal women. Moreover, drug resistance was associated with significantly higher levels of CA-125 (*p < 0.05*). The genes *UBA2*, *GLO1*, *STATH*, and *TUFT1* were differentiated in test samples from control samples. Moreover, drug resistance was associated with significantly higher expression of *GLO1*. The results of these assessments indicated the strong link between *UBA2* and hsa-miR-133a-3p and hsa-miR-133b; *GLO1* and hsa-miR-561-5p; *STATH* and hsa-miR-137-3p and hsa-miR-580-3p; and *TUFT1* and hsa-miR-1233-3p and hsa-miR-2052. Correlation analysis showed a significant correlation between CA-125 and HE4 levels. Moreover, a significant correlation between TUFT1 mRNA and UBA2, GLO1, STATH (negative correlation), and TUFT1 in relation to CA-125 and HE4 (*p* < 0.05) was noted in all patients. In view of the lack of screening tests for ovarian cancer, the occurrence of the described correlation may be inscribed as an attempt to establish an assay that meets the criteria of a screening test and thus increase the early diagnosis of ovarian cancer.

## Introduction

The incidence of ovarian cancer during a woman’s lifetime is estimated to be approximately 1 in 75 women, and mortality from the disease is nearly 1 in 100 women (1). Worldwide, ovarian cancer ranks fourth in terms of death due to malignancy, and it accounts for 5% of all cancers diagnosed in women and 31% of all cancers of the female reproductive system (2, 3). Unfortunately, it is also associated with the highest mortality rate among all gynecologic cancers (4). According to The American Cancer Society, it is estimated that 19,880 women will be diagnosed with ovarian cancer in 2022 (5). It should be kept in mind that ovarian cancer ranks fifth in all deaths associated with cancer in women and it has the highest risk of death compared with that in all gynecologic cancers (6, 7).

The risk of ovarian cancer increases with age. In the European continent, approximately 80% of ovarian cancers develop in women above the age of 50 years, most commonly between 60 and 64 years of age and after 75 years (8, 9). The risk of developing ovarian cancer increases in women who achieve menopause at a later age compared with those who reach it at an earlier age (10, 11). Important risk factors include infertility (generally no offspring), infertility if treated with ovulation induction, and recurrent inflammatory conditions including endometriosis, overweight/obesity, and smoking (1, 12–15).

According to the World Health Organization classification, primary ovarian cancers are divided into three groups: surface epithelial-stromal tumors, sex cord-stromal tumors, and germ cell tumors. Considering the molecular basis and clinical implications of tumorigenesis, two types of ovarian cancer can be distinguished. The first one develops from benign ovarian tumors or borderline tumors and constitutes approximately one-third of all cases. Type I includes serous carcinoma G1/2, endometroid carcinoma G1/2, mucinous carcinoma, clear cell carcinoma, and Brenner’s carcinoma. It is characterized by slow growth and low sensitivity to chemotherapy with a good prognosis (nearly 80% 5-year survival rate) and lower frequency of recurrence. Type II occurs significantly more often (70% of all cases) and is known to have a poor prognosis. It includes serous carcinoma G3, endometroid carcinoma G3, undifferentiated carcinoma G3, and sarcoma. It is most often diagnosed at stages III and IV and is characterized by rapid growth and high sensitivity to chemotherapy but with more frequent recurrence and poor prognosis (nearly 90% of patients die within 5 years of observation) (16–18).

Thus far, annual screening including transvaginal ultrasound and carcinoma antigen (CA-125) evaluation has not been proven to affect population-based detection of ovarian cancer. Additionally, computed tomography (CT), magnetic resonance imaging, and positron emission tomography are used to assess disease progression, monitoring treatment effects, and detecting recurrence (19, 20).

Among useful biochemical markers, CA-125 antigen, which is a glycoprotein that is not present in the epithelial cells of normal ovaries, and human epididymis protein 4 (HE4). The Risk of Ovarian Malignancy Algorithm (ROMA) is calculated based on the determined concentrations of the two tumor markers CA125 and HE4 and considering the woman’s menopausal status. On this basis, women can be classified into a high- or low-risk group for developing ovarian cancer. Performing the determinations of both markers simultaneously and calculating the ROMA values increases the diagnostic value of these tests. The role of ROMA in detecting early-stage ovarian cancer is particularly emphasized. The lower limit of normal for laboratory determinations is usually <35 IU/mL. CA-125 levels >35 IU/mL are observed in 50–90% of patients with ovarian cancer. Before surgical treatment, normal levels of CA-125 are found in 50% of women with stage I cancer and 60% women with stage II cancer (21, 22). The HE4 marker, found in the epithelium of the epididymis, trachea, salivary glands, lungs, kidneys, prostate, fallopian tubes, oral mucosa, endometrium, and endocervix, whose normal concentration is <150 pM/L, is also important. Because HE4 does not have such a high tendency for false-positive results, its determination is useful for clinical diagnosis. Elevated levels of HE4 are observed in cases of endometrial cancer, cervical cancer, and benign ovarian tumors. It should be noted that these markers are not specific and also change in situations not associated with cancer, such as endometriosis, pregnancy, or menstruation (23, 24).

The extent of treatment—surgery and possible supplementation with chemotherapy—depends on the disease stage. Unfortunately, in approximately one-fifth of cases, drug resistance to the platinum compounds (cisplatin) used as first-line chemotherapy is noted (25, 26).

The present study aimed to evaluate the changes in expression patterns at the gene and protein levels associated with the phenomenon of drug resistance, as well as the levels of CA-125 and HE4 markers and the association between them, in patients with stage I-IV ovarian cancer in comparison with control patients.

## Patients and methods

The present study was performed in accordance with the guidelines of the 2013 Declaration of Helsinki on human experimentation. It is not possible to identify patients on an individual basis either in this study or in the database. Informed consent was obtained from all patients. Approval from the Bioethical Committee operating at the Regional Medical Chamber in Kraków (approval no. 185/KBL/OIL/2020 and 186/KBL/OIL/2020, dated September 20, 2020) was obtained for this study.

### Patients

The study group included 48 women who had a histopathologically confirmed diagnosis of stage I-IV ovarian cancer, from which two subgroups (groups A and B) were identified.

In group A, there were 36 patients in whom surgical treatment was supplemented with first-line chemotherapy according to current standards. Within this patient group, 5 had stage I (14%), 5 had stage II (14%), 25 had stage III (69%), and 1 had stage IV ovarian cancer (3%). Loss of response to chemotherapy in this patient group was assessed on the basis of imaging examinations with CT, performed at intervals compliant with the current recommendations of the Response Evaluation Criteria in Solid Tumors (RECIST) (27, 28). Out of the 36 women in group A, drug resistance was found in 24 patients (67%), after cycle three of chemotherapy 17 patients displayed drug resistance (71%), including 2 women with stage II (12%), 14 women with stage III (82%) and 1 patient with ovarian cancer stage IV (6%) and after cycle six 7 patients displayed drug resistance (29%), including 1 patient with stage II (14%) and 6 patients with ovarian cancer stage III (86%).

Group B included 12 patients with type I ovarian cancer, including 11with stage I and 1 patient with stage IV, whose oncological treatment required only surgery. Chemotherapy was not necessary owing to the low staging of the neoplastic lesions. The control group (C) comprised of 50 women in whom the uterus and adnexa were surgically removed for non-oncological reasons.

Oncological treatment—surgical procedure including removal of the uterus with adnexa, appendix, mesh (non-mesh), pelvic minor lymph nodes, and pelvic and pre-aortic minor lymph nodes, as well as chemotherapy with cisplatin—was performed in the Gynecology and Obstetrics Department with Gynecology Oncology and Clinical Oncology Unit of Ludwik Rydygier Specialist Hospital in Kraków, Poland. Platinum resistance was defined as the recurrence of disease within 6 months after the completion of chemotherapy.

The detailed clinical characteristics of the patients are presented in **Table 1**. Patients treated with surgery and chemotherapy (group A) were significantly older, and their initial body weight and body mass index (BMI) were significantly lower than those of women in the other groups. Moreover, ascites and menopause were more frequent. A significant decrease in body weight under the influence of treatment was present in all the study groups (*p* < 0.05).

**Table 1:** Clinical and anthropometric data of patients with histopathologically confirmed diagnosis of stage I-IV ovarian cancer (groups A and B) and control subjects (group C).

Group		Test		Control	Total N=98 (100)	p-value
		A n=36 (36.7)	B n=12 (12.2)	C n=50 (51.0)		
Stage	I	5 (13.9)	11 (91.7)	0 (0)	16 (16.3)	–
	II	5 (13.9)	0 (0)	0 (0)	5 (5.1)
	III	25 (69.4)	0 (0)	0 (0)	25 (25.5)
	IV	1 (2.8)	1 (8.3)	0 (0)	2 (2)
Chemotherapy	No	0 (0)	12 (100)	0 (0)	12 (12.2)	–
	Yes	36 (100)	0 (0)	0 (0)	36 (36.7)
Chemotherapy resistance	Yes	24 (66.7)	–	–	–	–
Ascites	No	14 (38.9)	11 (91.7)	50 (100)	75 (76.5)	p<0.001^6^
	Yes	22 (61.1)	1 (8.3)	0 (0)	23 (23.5)
Menopause	No	6 (16.7)	6 (50)	39 (78)	51 (52)	p<0.001^6^
	Yes	30 (83.3)	6 (50)	11 (22)	47 (48)
Age [years]		63 (53-69.5)	47.5 (40-58.5)	46.5 (44-50)	50 (45-60)	p<0.001^2^ A vs B** A vs C***
Age at menopause [years]		50 (50-54)	51.5 (50-53)	50 (49-52)	50 (50-53)	0.98^2^
Height [cm]		160 (155-167)	162.5 (150-166)	158.5 (154-163)	159 (155-165)	0.58^2^
Body weight [kg]	before surgery (1)	62.9±10.7	63.5±8.3	69.9±10	66.6±10.6	0.005^1^ A vs C**
	after surgery (4 weeks) (2)	60.1±10.3 59 (52-69.5)	61.6±8 60.5 (54-69)	67.7±10 67 (61-76)	64.2±10.5 65 (55-71)	0.002^2^ A vs C**
	before chemotherapy (3)	56.6±9.4	–	–	56.6±9.4	–
	p-value	p<0.001^4^ 1 vs 2*** 2 vs 3*** 1 vs 3***	0.002^3^	p<0.001^3^	p<0.001^4^ 1 vs 2*** 2 vs 3*** 1 vs 3***	–
BMI [kg/m^2^]	before surgery (1)	24.5±4.4	25.1±4	27.6±4.5	26.1±4.7	0.003^1^ A vs C**
	after surgery (4 weeks) (2)	23.5±4.4	24.4±4.1	26.8±4.5	25.2±4.7	0.001^1^ A vs C**
	before chemotherapy (3)	21.8±4.1	–	–	21.8±4.1	–
	p-value	p<0.001^4^ 1 vs 2 vs 3*** 1 vs 3***	0.003^3^	p<0.001^3^	p<0.001^4^ 1 vs 2*** 2 vs 3*** 1 vs 3***	–

(A) ovarian cancer patients treated with surgery and chemotherapy; (B) ovarian cancer patients treated with surgery; (C) control group; BMI, body mass Index

Measurable data are presented as mean ± standard deviation or as media– with quartiles (Q1-Q3) depending on the form of distribution; p-value for groups - significance level with ANOVA^1^/Kruskal-Wallis^2^ test; p-value for repeated measures - significance level with Student’s t-test^3^/ANOVA^4^ For repeated measures. Non-measurable data are presented a’ number and percentage. p-value for groups - significance level with Chi-2^5^/Fisher’s exact test^6^; ** p < 0.01 determined by the post hoc test; *** p < 0.001 determined by the post hoc test.

### Materials

Tissue material collected during surgery was secured for molecular analyses in Allprotect Tissue Reagent (Qiagen, Wroclaw, Poland, Cat No./ID: 76405) in an Eppendorf tube and stored at -20°C until molecular analyses.

Blood samples were collected from the vein of the ulnar fossa from women in the study and control groups; the samples were collected into tubes designated for clotting, after which the samples were centrifuged for 10 minutes, at 1,500 ×g, at 20°C to obtain serum for further biochemical analyses. The samples were stored at -20°C until the start of the analysis.

### Evaluation of CA-125 and HE4 levels

Changes in the concentrations of CA-125 and HE4 markers were assessed from the serum of patients in the pretreatment and control groups by immunohistochemistry analysis involving electroluminescence detection (immunochemical analyzer Cobas e-411 Rack, Roche Diagnostics, Warsaw, Poland). The concentrations of these markers were determined based on a solid phase antigen-antibody reaction. The samples were incubated twice, first with biotinylated monoclonal antibodies specific for CA-125 (SigmaAldrich, Poznan, Poland, Catalog no. RAB0376-1KT) and HE4 (Abcam, Cambridge, MA, USA, Cat. No. ab132299), labelled with ruthenium complexes, and second with streptavidin-labeled microparticles. The reaction mixture was then transferred to the measuring chamber, where the microparticles were magnetically attracted to the electrode surface. Subsequently, unbound substances were removed using ProCell system fluids. The voltage that was applied to the electrode induced an electrochemiluminescence reaction and photon emission, which was measured using a photomultiplier. The results were read by constructing a two-point calibration curve.

### Extraction of the total RNA

Extraction of total RNA was performed using TRIzol reagent (INvitrogen Life Technologies, Carlsbad, CA, USA, Cat. no. 15596026) according to the manufacturer’s protocol. The isolated RNA was then evaluated qualitatively by performing electrophoretic separation of the extracts in 1% agarose gel and quantitatively by performing spectrophotometric analysis (NanoDrop ND, Thermo Fisher Scientific, Waltham, MA, USA). For further molecular analysis, only those RNA extracts that fulfilled the following conditions were qualified: 18S rRNA and 28S rRNA strands visible in the agarose gel, and the absorbance ratio at 260 nm to 280 nm was 1.8-2.0 in the quantitative evaluation.

### mRNA microarray analysis

The microarray profile of mRNA expression changes that are associated with drug resistance was determined using the HG-U133A 2_0 microarray (Affymetrix, Santa Clara, CA, USA), the GeneChip™ 3′ IVT PLUS Reagent Kit, and GeneChip™ HT 3′ IVT PLUS Reagent Kit (Thermo Fisher Scientific, Waltham, MA USA, Cat. no. 902416) according to the manufacturer’s recommendations. The mRNA names and their ID number were determined from the Affymetrix NetAffx™ Analysis Center database after entering the phrase “drug resistance” (http://www.affymetrix.com/analysis/index.affx; accessed on February 2, 2022). Data were analyzed using a microarray scanning GeneArray scanner (Agilent Technologies, Santa Clara, CA, USA).

### miRNA transcriptome analysis

Changes in the miRNA transcriptome in the test samples in comparison with the control samples were determined using the miRNA microarray technique GeneChip miRNA 2.0 Array (Affymetrix), as described by the manufacturer. Data were analyzed using a microarray scanning GeneArray scanner (Agilent Technologies).

Predictive evaluation of the effect of selected miRNAs on mRNAs were significantly differentiated in test samples from control samples was performed using the TargetScan database (http://www.targetscan.org; accessed on February 15, 2022) (29) and miRanda (http://mirdb.org; accessed on15 February 2022) (30, 31). According to the miRDB database, “This is an online database for miRNA target prediction and functional annotations. All the targets in miRDB were predicted using a bioinformatics tool, MirTarget, which was developed by analyzing thousands of miRNA-target interactions from high-throughput sequencing experiments. Common features associated with miRNA binding and target downregulation have been identified and are used to predict miRNA targets with machine learning methods. A predicted target with a prediction score of >80 is most likely to be real; however, if the score is below 60, then one needs to exercise caution, and it is recommended to have other supporting evidence as well” (30, 31).

### Reverse-transcription quantitative polymerase chain reaction assay

To validate the semi-quantitative results of the microarray expression pattern of the mRNAs evaluated, reverse-transcription quantitative polymerase chain reaction (RT-qPCR) was performed using the SensiFast ™ SYBR No-ROX One-Step Kit (Bioline, London, UK), where β-actin was used as the endogenous control.

The thermal profile of the reaction was as follows: reverse transcription (45°C for 10 min); activation of the polymerase (95°C for 2 min); and 40 cycles of denaturation (95°C for5 s), annealing (60°C for 10 s), and elongation (72°C for 5 s). In **Table 2** the nucleotide sequence of primers are presented.

**Table 2:** Nucleotide sequences of primers used to amplify genes differentiated in the study samples (group A and B) from the control samples (group C) by the RT-qPCR.

mRNA	Primer sequence (Forward, reverse)
*UBA2*	Forward 5’-AAAAAGGGTGTGACCGAGTG-3’ Reverse 5’-GCATCTTCTTCCCCAAACAA-3’
*GLO1*	Forward 5’-GCGTAGTGTGTGACTCCT-3’ Reverse 5’-TCACTCGTAGCATGGTCTGC-3’
*STATH*	Forward 5’-TTTGCCTTCATCTTGGCTCT-3’ Reverse 5’-TGTGGTTGGTATGGTTTGG-3’
*TUFT1*	Forward 5’-TCAGTCATGGCAACTTCAGC-3’ Reverse 5’-GGGACAGTCAGGAAGTCAA-3’

UBA2, ubiquitin-like modifier-activating enzyme 2; GLO1, glyoxalase I; STATH, statherin; TUFT1, tuftelin 1; RT-qPCR, reverse-transcription-polymerase chain reaction.

Changes in gene expression were evaluated with the relative method for assessing gene transcriptional activity (also known as 2^-ΔΔCT^).

### Enzyme-linked immunosorbent assay

After rinsing the slides with phosphate-buffered saline (PBS) solution to remove blood residues, the tissue samples were mechanically homogenized in PBS (10 mg tissue per 100 μL PBS) and centrifuged for 15 min at 1,500 ×g. After collecting the supernatant, enzyme-linked immunosorbent assay (ELISA) was immediately performed.

To determine the concentration of the analyzed proteins, we used the UBA2 Elisa kit (Human ubiquitin-like modifier-activating enzyme 2 ELISA Kit, MyBioSource, Inc. San Diego, CA 92195-3308, USA, Cat. no. MBS9317388), Human GLO1 ELISA Kit (MyBioSource, Inc., Inc., San Diego, CA, USA,Cat. no. MBS761164), Human GLO1 ELISA Kit (MyBioSource, Inc., Inc., San Diego, CA, USA, Cat. no. MBS2533426), and TUFT ELISA kit (Human Tuftelin (TUFT) ELISA Kit, MyBioSource, Inc., Inc., San Diego, CA, USA, Cat. no. MBS2104898) in accordance with the manufacturer’s recommendation.

### Statistical analysis

Statistical analysis of data obtained in the mRNA microarray analysis was performed using the Transcriptome Analysis Console program (Thermo Fisher Scientific, Waltham, MA, USA) which links the CEL file analysis and QC features of Expression Console and the statistical analysis of TAC into a single software application.” In the first step, the results were normalized using the Robust Multiarray Average (RMA) method, which consisted in the logarithmic transformation of the fluorescence signal value for each transcript (log_2_). Based on the log_2_ Fold Change (FC) value, the multiple of the difference between the expression level of mRNA transcriptomes of the compared groups was assessed, while the statistical strength of the observed difference was assessed based on the *p*-value. The criterion for recognizing a gene as differentiating required that the absolute value of the difference in fluorescence signals between the compared groups (FC) was greater than 1.1 (minimum 1.1-fold decrease or increase in signal intensity) and the *p* value < 0.05.

Data analysis was performed using the Statistica 13.3 program (Stat Soft, Poland) and R, version 4.1.1 (The R Foundation for Statistical Computing).

For non-measurable data, numeric-percentage notation was used and χ^2^ or Fisher’s exact test of independence was employed. Measurable data were presented as mean ± standard deviation (SD) and median with quartiles (Q1-Q3). Compliance with normal distribution was verified with the Shapiro-Wilk test.

If data were normally distributed, we used Student’s t-test (comparison of the two groups) or one-way analysis of variance ANOVA with Bonferroni correction and *post-hoc* Tukey’s honestly significant difference test (comparison of more than two groups) to determine the statistically significant differences in mean values. In turn, if data were skewed statistically significant differences in distributions were analyzed using Mann–Whitney U (comparison of the two groups) or Kruskal–Wallis’s test with Bonferroni correction (comparison of more than two groups) and *post-hoc* Dunn’s test or Scheirer–Ray–Hare test (non-parametric version of two-way ANOVA based on ranks).

Correlation analysis was performed using Spearman’s correlation coefficient and its significance test. Moreover, odds ratios (OR) with their confidence intervals (Cis) were determined using univariate logistic regression models. Multivariate analysis did not identify a significant regression model. When interpreting the results, a *p*-value of < 0.05 was considered as indicating statistical significance.

## Results

### Concentration of the markers CA-125 and HE4

We first evaluated changes in the concentrations of the two biochemical markers currently used in diagnosis: CA-125 and HE4. Significantly higher levels of CA-125 and HE4 were observed in group A and among menopausal women. Moreover, drug resistance was associated with significantly higher CA-125 levels (**Table 3**; *p* < 0.05).

**Table 3:** Concentrations of CA-125 and HE4 in groups of patients with histopathologically confirmed diagnosis of ovarian cancer (groups A and B) and control group (C), including patients undergoing chromotherapy (in A group) and menopause (total and in A group).

Group	Total N=98 (100)	Test		Control	p-value
		A n=36 (36.7)	B n=12 (12.2)	C n=50 (51.0)	
CA-125	5.3 (0.6-144.9)	320 (65-751.2)	21.4 (7.9-79)	0.6 (0.3-1)	p<0.001^2^ A vs C*** B vs C***
HE4	72 (44-439)	439 (243-772.5)	83.1 (61.9-302)	44.9 (32.4-54.9)	p<0.001^2^ A vs C*** B vs C*
Indicators		Ca-125		HE4	
Chemo-therapy resistance Group A	Yes	563.8 (166.7-1545.2)		485.5 (238.5-772.5)	
	No	75.5 (27.5-320)		298.5 (243.5-662.5)	
p-value		0.04^1^		0.61^1^	
Indicators		Before menopause	Menopause	Before menopause	Menopause
Total		0.9 (0.4-3)	79 (7-588.2)	47.1 (33.8-65.3)	289 (120-637)
p-value		p<0.0001^1^		p<0.0001^1^	
Chemo-therapy resistance Group A	Yes	50.6 (44-397)	588.2 (167-1821.1)	167 (98-332.5)	554.5 (289-838.5)
	No	315 (315-315)	45 (13-325)	1176.5 (482-1871)	264.5 (235-324)
p-value		0.15^3^		0.07^3^	

(A) ovarian cancer patients treated with surgery and chemotherapy; (B) ovarian cancer patients treated with surgery; (C) control group; ANOVA, analysis of variance

Measurable data are presented as mean ± standard deviation or as median with quartiles (Q1-Q3) depending on the form of distribution; p-value for groups - significance level with U Mann-Whitney^1^/Kruskal-Wallis^2^ test; p-value in 2-factor analysis - significance level with Scheirer-Ray-Hare^3^ test; * p < 0.05 determined by the post hoc test; p < 0.01 determined by the post hoc test.

### Microarray analysis

In the first stage of microarray analysis mRNAs that were significantly differentiated in ovarian cancer samples from control samples (*p*<0.05) were selected. Out of the 47 mRNAs associated with drug resistance, 12 mRNAs were significantly differentiated in the study samples (group A and B) from control samples (one-way ANOVA variance analysis; *p*<0.05).

**Figure 1:**
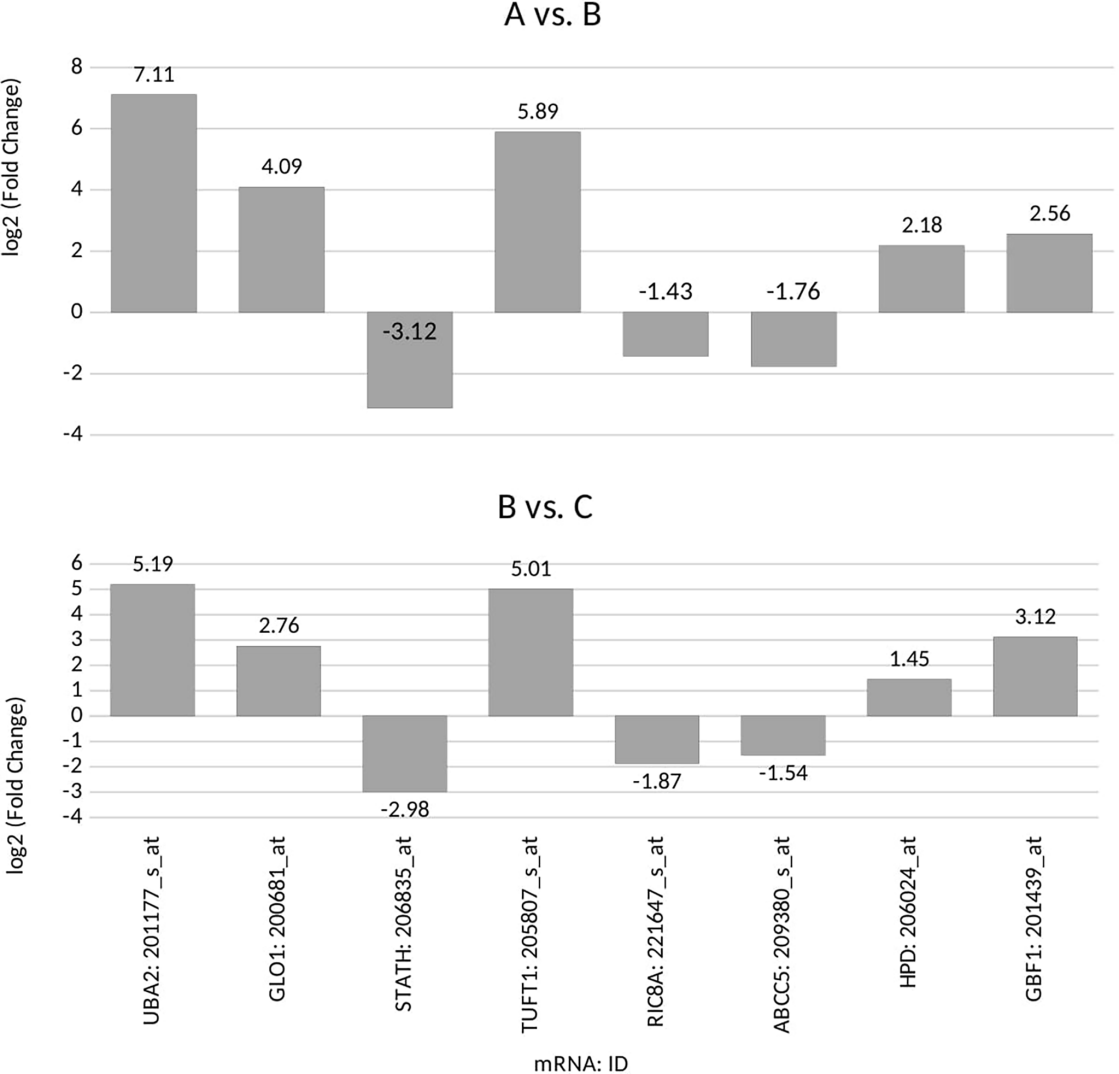
Microarray expression profile of genes associated with drug resistance differentiating between the ovarian cancer samples (groups A and B) from control samples (group C) *(p*<0.05) for: *UBA2*, ubiquitin-like modifier-activating enzyme 2; *GLO1*, glyoxalase I; *STATH*, statherin; TUFT1, tuftelin 1; *RIC8A*, RIC8 guanine nucleotide exchange factor B; *ABCC5*, ATP-binding cassette subfamily C member 5; *HPD*, 4-hydroxyphenylpyruvate dioxygenase; *GBF1*, Golgi brefeldin A-resistant guanine nucleotide exchange factor 1; (+), overexpression in comparison with the control (–);, downregulation in comparison with the control.

According to the *post-hoc* Tukey’s honestly significant difference test, we observed that seven genes, *UBA2*, *GLO1*, *STATH*, *TUFT1*, *RIC8A*, *ABCC5*, and *HPD*, were differentiated in group A vs. C samples. Five genes, *UBA2*, *GLO1*, *STATH*, *TUFT1*, and *GBF1*, were differentiated in group B vs. C samples. Four genes, *UBA2*, *GLO1*, *STATH*, and *TUFT1.* were common to group A and B (**Figure 1**; *p* < 0.05).

### RT-qPCR assay

In the next step, we quantified the expression of the four common genes differentiated in ovarian cancer samples from control samples. The same direction of expression changes of the selected transcripts by the RTqPCR technique, as in microarray analysis, was noted. We noted that the mRNA of *UBA2, GLO1, TUFT1, HPD*, and *GBF1* were upregulated in the ovarian cancer samples in comparison to the control samples (**Table 4**). The mRNA of *STATH, RIC8A*, and *ABCC5* levels were downregulated in cancer samples in comparison to the control samples (**Table 4**). Significantly higher absolute mRNA values, *UBA2, GLO1, STATH*, and *TUFT1*, were observed in group A (**Table 4**). Moreover, drug resistance was associated with significantly higher *GLO1* expression in group A (*p* < 0.05). Significantly higher mRNA values of *GLO1* were observed among women undergoing chromotherapy (in A group) and menopause (total and in A group) (*p* < 0.05).

**Table 4:** Expression pattern of selected genes in the study groups (A and B) in comparison with the control group (C) obtained by RT-qPCR, including patients undergoing chromotherapy (in A group) and menopause (total and in A group).

mRNA	Total N=98 (100)	Comparison group				Control		p-value	
		A vs. C n=36 (36.7)		B vs. C n=12 (12.2)		C n=50 (51.0)			
*UBA2*	8.9 (6.3-9.4)	9.2 (8.4-9.7)		6.1 (5.9-6.9)		–		0.0002^2^	
*GLO1*	3.5 (2.8-4)	3.8 (3.2-4.1)		1.9 (1.7-2.1)		–		p<0.0001^2^	
*STATH*	-3.4±0.7	-3.5±0.6		-2.9±0.6		–		0.004^1^	
*TUFT1*	5.2 (4.9-7)	6.3 (5.1-7.7)		4.1 (4.1-4.6)		–		p<0.0001^2^	
mRNA		UBA2		GLO1		STATH		TUFT1	
Chemo-therapy resistance Group A	Yes	9.1 (7.6-9.8)		3.9±0.4		-3.5±0.6		6.5±1.6 6.4 (5.2-7.7)	
	No	9.2 (8.9-9.4)		3.5±0.6		-3.4±0.7		6.3±1.9	
p-value		0.99^2^		0.02^1^		0.59^1^		0.83^1^	
mRNA		Before menopause	Menopause	Before menopause	Menopause	Before menopause	Menopause	Before menopause	Menopause
Total		7.6 (6.1-9.1)	9 (6.8-9.7)	2.8±1	3.5±0.8	-3.2±0.8	-3.4±0.6	5.9±2	5.9±1.7
p-value		0.27^2^		0.02^1^		0.37^1^		0.91^1^	
Chemotherapy resistance Group A	Yes	9 (8.5-10.1)	9.2 (7.1-9.8)	4 (3.9-4.1)	3.9 (3.6-4.1)	-3.9 (-4--3.8)	-3.4 (-3.9--3.1)	7.5 (6.6-8)	6.2 (5.2-7.3)
	No	9.1 (9-9.2)	9.3 (8.9-9.5)	3.1 (3.1-3.2)	3.4 (3.1-4.1)	-3.2 (-3.2--3.1)	-3.4 (-4--3)	7.7 (5.1-10.3)	5.8 (5.1-7)
p-value		0.84^3^		0.19^3^		0.12^3^		0.92^3^	

(A) ovarian cancer patients treated with surgery and chemotherapy; (B) ovarian cancer patients treated with surgery; (C) control group; ANOVA, analysis of variance; RT-qPCR, reverse-transcription-polymerase chain reaction

UBA2, ubiquitin-like modifier-activating enzyme 2; GLO1, glyoxalase I; STATH, statherin, TUFT1, tuftelin 1; (+), overexpression in comparison with the control; (-), downregulation in comparison with the control group.

Measurable data are presented as mean ± standard deviation and median with quartiles (Q1-Q3) depending on the form of distribution; p-value for groups - significance level with t-Student^1^/U Mann Whitney^2^ test; p-value in 2-factor analysis - significance level with Scheirer-Ray-Hare^3^ test

### Expression pattern of selected miRNAs

**Figure 2:**
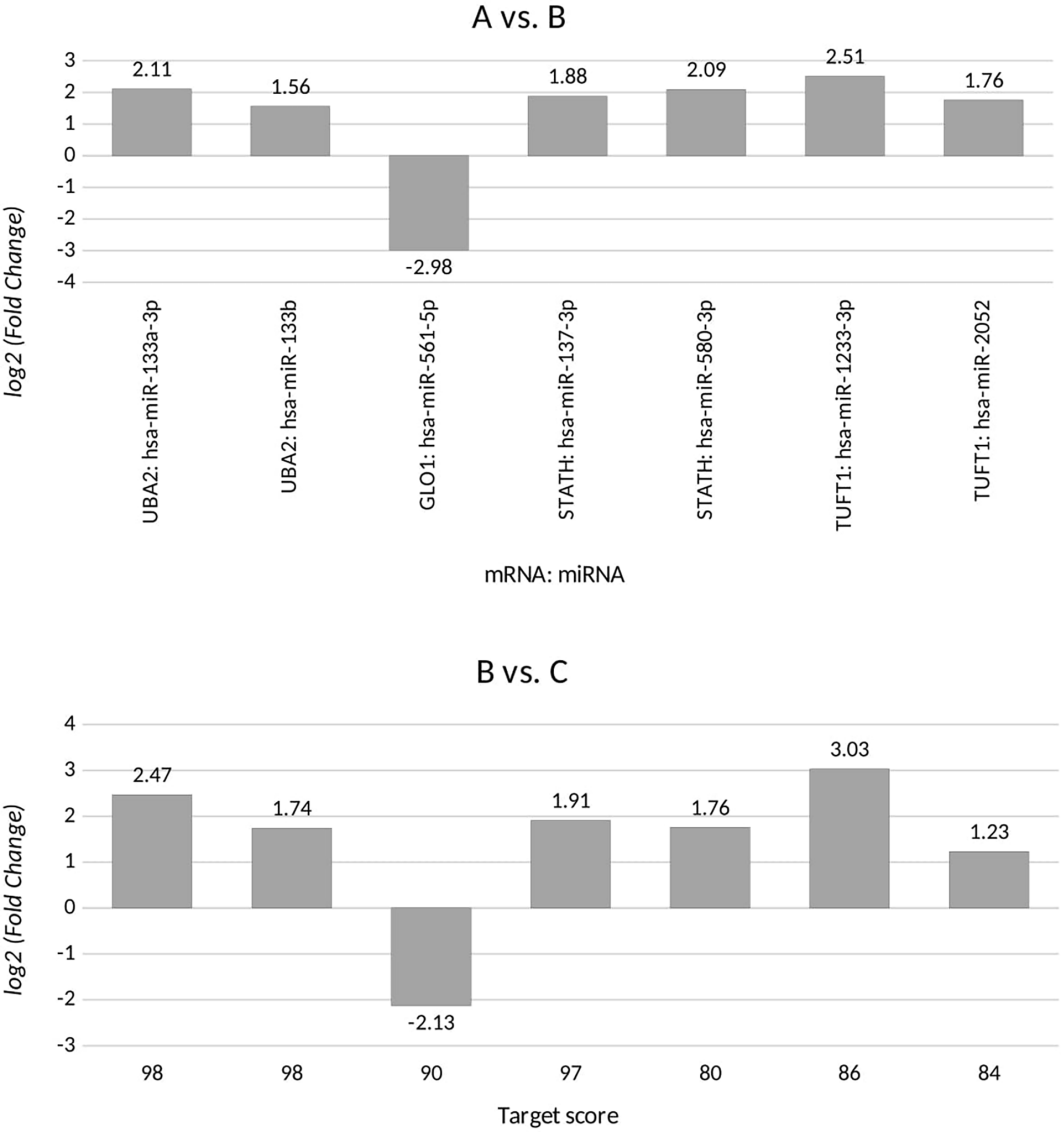
Changes in miRNA expression levels for differentiating ovarian cancer samples (groups A and B) from control samples (group C) that are potentially involved in regulating the expression of the selected transcripts, for *UBA2*, ubiquitin-like modifier-activating enzyme 2; *GLO1*, glyoxalase I; *STATH*, statherin, *TUFT1*, tuftelin 1; *RIC8A*, RIC8 guanine nucleotide exchange factor B; *ABCC5*, ATP-binding cassette subfamily C member 5; *HPD*, 4-hydroxyphenylpyruvate dioxygenase; *GBF1*, Golgi brefeldin A-resistant guanine nucleotide exchange factor 1. (+), overexpression in comparison with the control; (-), downregulation in comparison with the control.

**Figure 3:**
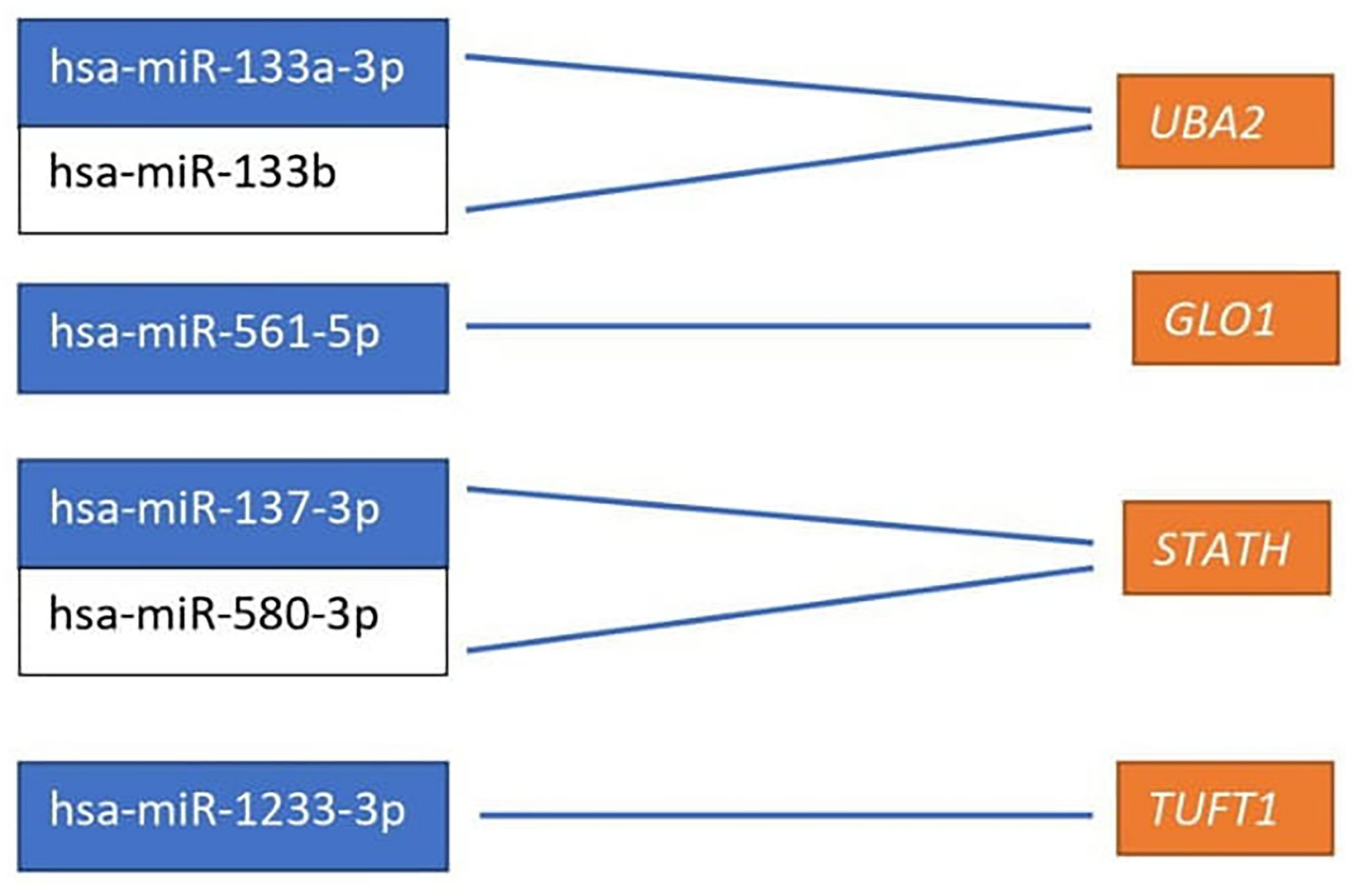
miRNAs affecting the transcriptional activity of genes differentiating ovarian cancer samples compared to the control samples. *UBA2*, ubiquitin-like modifier-activating enzyme 2; *GLO1*, glyoxalase I; *STATH*, statherin, *TUFT1*, tuftelin 1; *RIC8A*, RIC8 guanine nucleotide exchange factor B; *ABCC5*, ATP-binding cassette subfamily C member 5; *HPD*, 4-hydroxyphenylpyruvate dioxygenase; *GBF1*, Golgi brefeldin A-resistant guanine nucleotide exchange factor 1.

Based on the target score value, we observed the strongest link between the following entities: *UBA2* and hsa-miR-133a-3p (target score 98) and hsa-miR-133b (target score 98); *GLO1* and hsa-miR-561-5p (target score 90), *STATH* and hsa-miR-137-3p (target score 97) and has-miR-580-3p (target score 80); *TUFT1* and hsa-miR-1233-3p (target Ire 86), and hsa-miR-2052 (target score 94). It was observed that only one miRNA corresponding to hsa-miR-561-5p was downregulated in ovarian cancer samples in comparison to the control group (*p* < 0.05). For the remaining miRNAs, we found overexpression in the ovarian cancer samples compared to the controls (*p* < 0.05). In addition, we determined the same direction of change in expression in both groups of ovarian cancer samples (*p* < 0.05). Changes in the expression of the indicated miRNAs in individual groups of women with ovarian cancer in comparison with control subjects are shown in **Figure 2**. Then, with the use of bioinformatics tools, it was shown, which miRNAs are potentially involved in the regulation of the expression of previously selected mRNAs (**Figure 3**).

### ELISA results

Significantly higher absolute levels of UBA2, GLO1, and TUFT1 proteins were observed in group A and among menopausal women (**Table 5**; *p <* 0.0001). Notably, the *STATH* protein level was significantly higher in group B (*p <* 0.0001) and among premenopausal women (*p <* 0.0001). No significant difference was found in relation to the existence of drug resistance.

**Table 5:** Differences in the concentration of UBA2, GLO1, TUFT1, and STATH in the study (A and B) and control (C) groups obtained by ELISA assay, and their relationship with chemotherapy resistance including patients undergoing chromotherapy (in A group) and menopause (total and in A group).

Protein	Total N=98 (100)	Test				Control		p-value	
		A n=36 (36.7)		B n=12 (12.2)		C n=50 (51.0)			
UBA2	2 (0.9-4.2)	4.5 (4.1-4.9)		2.2 (2.2-2.6)		0.9 (0.5-1.1)		p<0.0001^3^ A vs B* A vs C*** B vs C**	
GLO1	5.3 (3.4-55)	56.6 (55-58.4)		14.2 (12.8-15)		3.4 (3.2-3.9)		p<0.0001^3^ A vs B* A vs C*** B vs C**	
STATH	19.1 (12.7-24.6)	12.3 (11.5-12.8)		99.6 (95.1-103.9)		23.6 (19.1-24.6)		p<0.0001^3^ A vs B*** A vs C*** B vs C**	
TUFT1	1 (0.6-12.5)	12.9 (11.9-13.9)		7.4 (7.2-7.8)		0.6 (0.6-0.8)		p<0.0001^3^ A vs B* A vs C*** B vs C**	
Protein		UBA2		GLO1		STATH		TUFT1	
Chemotherapy resistance Group A	Yes	4.5±0.5		57.4±2.5		12.2±0.9		13.3±1.6	
	No	4.4±0.5		54.9±4		12.2±0.9		12.8±1.1	
p-value		0.73^1^		0.07^1^		0.88^1^		0.32^1^	
Protein		Before menopause	Menopause	Before menopause	Menopause	Before menopause	Menopause	Before menopause	Menopause
Total		1.1 (0.8-2)	4.1 (1.9-4.8)	3.8 (3.2-5.3)	54.5 (12-57.8)	23.6 (18-24.8)	12.8 (11.9-24.5)	0.7 (0.6-1)	11.9 (7-13.5)
p-value		p<0.0001^2^		p<0.0001^2^		p<0.0001^2^		p<0.0001^2^	
Chemotherapy resistance Group A	Yes	4.2 (4-4.6)	4.5 (4.1-4.9)	58.7 (55.9-61.2)	57.3 (55.2-58.8)	12.2 (11.7-12.6)	12.3 (11.5-12.9)	13.8 (12.7-15.5)	12.9 (12-13.8)
	No	3.7 (3.3-4.1)	4.7 (4.2-4.9)	54.5 (54.2-54.9)	54.8 (51.2-57)	13.3 (13-13.6)	12.1 (11.5-12.7)	12.3 (11.8-12.8)	12.8 (11.8-13.8)
p-value		0.21^4^		0.4^4^		0.05^4^		0.24^4^	

(A) ovarian cancer patients treated with surgery and chemotherapy; (B) ovarian cancer patients treated with surgery; (C) control group.

ELISA, enzyme-linked immunosorbent assay; UBA2, ubiquitin-like modifier-activating enzyme 2; GLO1, glyoxalase I; STATH, statherin, TUFT1, tuftelin 1

Measurable data are presented as mean ± standard deviation or as median with quartiles (Q1-Q3) depending on the form of distribution; p-value for groups - significance level with Student’s t-test^1^/U Mann-Whitney^2^/Kruskal-Wallis^3^ test; p-value in 2-factor analysis - significance level with Scheirer-Ray-Hare^4^ test; * p < 0.05 determined by the post hoc test; ** p < 0.01 determined by the post hoc test; *** p < 0.001 determined by the post hoc test.

### Changes in the expression of the selected mRNA-miRNA-proteins

We assessed the relationship between the expression of mRNA, miRNA regulating each mRNA, and the respective protein coded for by the selected mRNA (**Table 6**). For *UBA2, GLO1*, and *STATH* in both groups (groups A and B), compared with the control group, we found the same level of expression changes at the mRNA and protein levels (up/up or down/down). However, when comparing the expression between groups B and C for *TUFT1*, a different expression profile at the mRNA and protein levels (up/down) was noted. It can be concluded that overexpression of an miRNA potentially regulating the expression of a given mRNA, only for the *STATH* and (hsa-miR-137-3p or hsa-miR-580-3p) resulted in silencing at the protein level. In turn, silencing the expression of hsa-miR-561-5p, potentially regulating *GLO1* mRNA expression, resulted in its overexpression at the transcript and protein levels in the test group compared to the control.

**Table 6:** Summarizing the changes in the expression of the selected mRNA-miRNA-protein for differentiating ovarian cancer samples (groups A and B) from control samples (group C).

Group	A vs. C			B vs. C		
Expression	mRNA	miRNA related to mRNA	Protein	mRNA	miRNA related to mRNA	Protein
*UBA2*	*up*	*up*	*up*	*up*	*up*	*up*
*GLO1*	*up*	*down*	*up*	*up*	*down*	*up*
*STATH*	*down*	*up*	*down*	*up*	*up*	*up*
*TUFT*	*up*	*up*	*up*	*down*	*up*	*up*

(up), overexpression in comparison with the control; (down), downregulation in comparison with the control; (A) ovarian cancer patients treated with surgery and chemotherapy; (B) ovarian cancer patients treated with surgery; (C) control group.

UBA2, ubiquitin-like modifier-activating enzyme 2; GLO1, glyoxalase I; STATH, statherin, TUFT1, tuftelin 1.

### Correlation analysis

Correlation analysis indicated a significant association between the levels of the CA-125 and HE4 markers (**Table 7**). Moreover, a significant association of *TUFT1* mRNA, as well as proteins UBA2, GLO1, STATH (negative correlation), and TUFT1 in relation to CA-125 and HE4 (*p* < 0.0001) was evident for all patients.

**Table 7:** Correlation between levels of biochemical and molecular markers, as values of Spearman’s R coefficients and the significance *p*-value, among women with ovarian cancer (groups A and B) compared with those of women in the control group (C).

Ca-125 vs.		Total N=98 (100)	Test		Control
			A n=36 (36.7)	B n=12 (12.2)	C n=50 (51.0)
HE4		0.84 p<0.001	0.45 p=0.02	0.72 p=0.02	0.34 p=0.055
mRNA	UBA2	0.22 p=0.2	-0.11 p=0.61	0.19 p=0.6	–
	GLO1	0.44 p=0.007	0.03 p=0.89	0.15 p=0.68	–
	STATH	-0.07 p=0.71	0.03 p=0.89	0.49 p=0.15	–
	TUFT1	0.51 p=0.001	0.13 p=0.54	0.44 p=0.2	–
Protein	UBA2	0.76 p<0.0001	0.29 p=0.14	0.07 p=0.85	-0.21 p=0.22
	GLO1	0.83 p<0.0001	-0.09 p=0.65	0.76 p=0.01	0.21 p=0.22
	STATH	-0.49 p<0.0001	-0.26 p=0.2	-0.05 p=0.88	-0.16 p=0.36
	TUFT1	0.8 p<0.0001	-0.13 p=0.52	0.2 p=0.58	-0.0008 p=0.996
Ca-125 vs.		mRNA-UBA2	mRNA-GLO1	mRNA-STATH	mRNA-TUFT1
Chemotherapy resistance	Yes	-0.08 p=0.75	-0.12 p=0.63	-0.3 p=0.23	-0.09 p=0.74
	No	-0.29 p=0.49	-0.4 p=0.32	0.69 p=0.06	0.38 p=0.35
Ca-125 vs.		Protein-UBA2	Protein -GLO1	Protein -STATH	Protein -TUFT1
Chemotherapy resistance	Yes	0.47 p=0.05	-0.27 p=0.28	-0.24 p=0.33	-0.37 p=0.13
	No	0.34 p=0.42	-0.69 p=0.06	-0.3 p=0.47	0.17 p=0.69
HE4 vs.		Total N=98 (100)	Test		Control
A n=36 (36.7)	B n=12 (12.2)	C n=50 (51.0)
mRNA	UBA2	0,13 p=0,38	-0,12 p=0,48	0,6 p=0,07	–
	GLO1	0,19 p=0,21	-0,14 p=0,43	-0,27 p=0,45	–
	STATH	0,23 p=0,13	0,34 p=0,04	0,72 p=0,02	–
	TUFT1	0,42 p=0,004	0,24 p=0,15	0,26 p=0,47	–
Protein	UBA2 [ng/mL]	0,72 p<0.0001	0,09 p=0,58	0,11 p=0,76	-0,08 p=0,65
	GLO1 [pg/mL]	0,74 p<0.0001	-0,15 p=0,37	0,76 p=0,01	0,04 p=0,79
	STATH [pg/mL]	-0,52 p<0.0001	0,05 p=0,77	-0,08 p=0,83	-0,04 p=0,82
	TUFT1 [pg/mL]	0,7 p<0.0001	-0,08 p=0,65	0,21 p=0,56	-0,24 p=0,15
HE4 vs.		MRNA-UBA2	MRNA-GLO1	MRNA-STATH	MRNA-TUFT1
Chemotherapy resistance	Yes	-0.28 p=0.19	-0.12 p=0.59	0.24 p=0.26	0.28 p=0.19
	No	0.29 p=0.35	-0.29 p=0.35	0.57 p=0.05	0.15 p=0.63
HE4 vs.		Protein-UBA2	Protein -GLO1	Protein STATH	Protein TUFT1
Chemotherapy resistance	Yes	0.14 p=0.53	-0.17 p=0.43	-0.04 p=0.85	-0.18 p=0.4
	No	-0.02 p=0.94	-0.14 p=0.66	0.33 p=0.29	0.09 p=0.78
mRNA vs.		mRNA-UBA2 +	mRNA-GLO1 +	mRNA-STATH -	mRNA-TUFT1 +
Protein	UBA2	0.32 p=0.03	0.57 p<0.0001	-0.35 p=0.01	0.42 p=0.003
	GLO1	0.46 p<0.0001	0.61 p<0.0001	-0.43 p=0.002	0.5 p<0.0001
	STATH	-0.56 p<0.0001	-0.58 p<0.0001	0.39 p=0.01	-0.49 p<0.0001
	TUFT1	0.57 p<0.0001	0.63 p<0.0001	-0.14 p=0.35	0.49 p<0.0001

(A) ovarian cancer patients treated with surgery and chemotherapy; (B) ovarian cancer patients treated with surgery; (C) control group.

UBA2, ubiquitin-like modifier-activating enzyme 2; GLO1, glyoxalase I; STATH, statherin, TYFT1, tuftelin 1.

The results of the analyses are presented as values of Spearman’s R coefficients together with the result of the significance test (p-value).

### Risk factors for drug resistance in patients with ovarian cancer and the occurrence of malignancy

**Figure 4:**
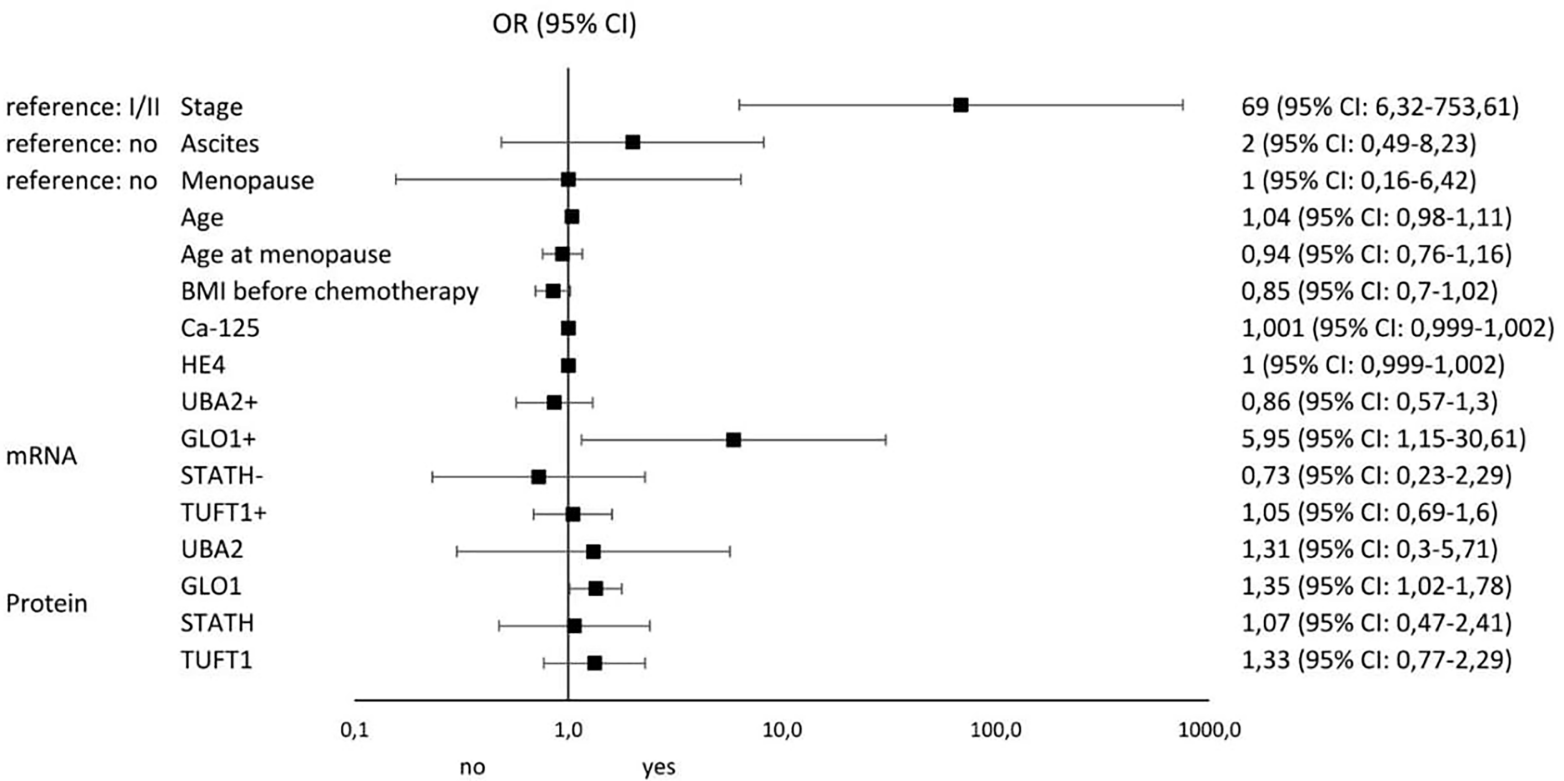
Results of univariable logistic regression analysis - odds ratio (OR) and its 95% confidence interval (95% CI) for the relationship between the occurrence of chemotherapy resistance (no/yes) and particular classification variables in group A of patients with histopathologically confirmed diagnosis of stage I-IV ovarian cancer, treated with surgery and supplemented with chemotherapy as per –tandard guidelines.

**Figure 5:**
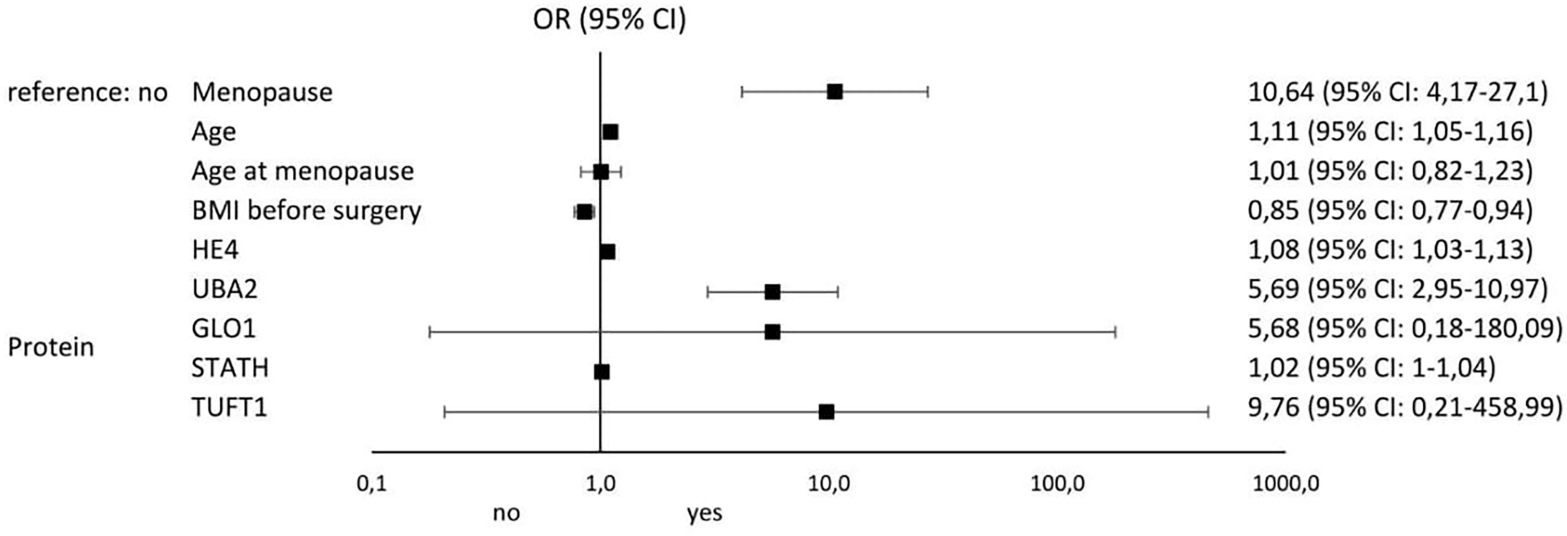
Results of univariable logistic regression analysis - odds ratio (OR) and its 95% confidence interval (95% CI) for the relationship between the occurrence of cancer (no/yes) and the particular classification variables of all patients in the study groups (A, B, and C).

**Figures 4** and **5** illustrate the influence of selected factors on the occurrence of drug resistance and cancer. A significantly higher risk of drug resistance in group A patients was observed in women with stage III/IV disease due to increased expression of *GLO1* mRNA and protein encoded by this gene. A significantly higher cancer risk was associated with menopause, age, lower BMI, and higher levels of HE4 and UBA2 protein.

## Discussion

Despite many years of research and the development of modern diagnostic techniques including biochemical and molecular analyses, ovarian cancer is diagnosed at very late stages and the available treatments do not provide the expected outcome, which is attributed to the development of drug resistance during chemotherapy (7, 32).

In our analysis, we evaluated changes in the levels of biochemical markers—CA-125 and HE4—and the expression profile of genes and proteins encoded by them associated with drug resistance in patients with ovarian cancer in comparison to control subjects. Significantly higher concentrations of these markers were found in the serum of patients in the study group, regardless of whether treatment was supplemented with chemotherapy or not. Higher levels of CA-125 and HE4 being characteristic of patients in group A, in whom resistance to cisplatin was observed, and in premenopausal age.

Potenza et al. evaluated 78 patients diagnosed with epithelial ovarian cancer regarding the utility of CA-125 and HE4 determination in monitoring response to cytotoxic treatment. The authors concluded that both parameters are good markers of loss of adequate response to treatment, as their levels were not recorded after the third cycle of chemotherapy in patients with an initially good response to treatment (33). Nevertheless, Castella et al., who also evaluated changes in CA-125 and HE4 levels among 72 patients with ovarian cancer, reported that an increase in biomarkers corresponded with disease recurrence, confirmed by diagnostic imaging, in most patients, although in some women, there was an upward trend in the CA-125 and HE4 levels not associated with recurrence (34). This leads to the search for new markers, mainly at the molecular level, on the basis of which the occurrence of drug resistance can be established before its phenotypic manifestation, because molecular changes precede phenotypic changes (35, 36). It is a more reasonable approach as we demonstrated significant correlation between CA-125 and HE4 levels and the four proteins that were differentiated in the test samples in groups A and B from control samples (*p* < 0.05). Studies on a larger population of patients should be performed to determine the usefulness of CA-125 and HE4 analyses and the mentioned proteins in the diagnosis of ovarian cancer and determination of resistance to chemotherapy.

In our study, we found drug resistance in 24 of 36 patients (66.7%) in whom surgery was supplemented with chemotherapy. Therefore, in the second stage of our study, we decided to assess the changes in the expression of genes related to the drug resistance phenomenon that were specifically differentiated in the study group from control samples and the miRNAs that potentially regulate their expression in groups A and B. Based on microarray evaluation of the collected samples, we showed that, in the cases of ovarian cancer, irrespective of the stage and selected treatment option, the expression profile of mRNAs and miRNAs were related to drug resistance changes. mRNAs corresponding to genes *UBA2*, *GLO1*, *STATH*, and *TUFT1*, were differentiated in the study samples, irrespective of the stage of ovarian cancer from the control samples, and we decided to focus on them in further analyses.

He at al. observed a significant increase in the gene and protein expression of *UBA2* in colorectal cancer samples when compared with control samples, identifying overexpression of this gene as an adverse prognostic marker (37). Moreover, UBA2 participates in the processes of tumor progression, invasion, and metastasis through the Wnt-dependent pathway, consequently promoting epithelial–mesenchymal transition (38). In our analysis, we demonstrated overexpression of *UBA2* mRNA, and the protein coded by it in ovarian cancer samples (*p* < 0.05), it was higher in group A where surgical treatment was complemented by chemotherapy when compared with group B where treatment was terminated by surgery. Nevertheless, we did not identify any relationship between cisplatin drug resistance and UBA2 expression (*p* > 0.05).

Our analysis also showed that *GLO1* mRNA were differentiated in study samples from control samples and is the only transcript among the four mRNAs selected for which we could identify a significant association with the occurrence of cisplatin drug resistance (OR = 5.95; 95% CI 1.15-30.61; *p =* 0.02). Among patients who lost adequate response to treatment, *GLO1* expression was significantly higher than that among women with ovarian cancer responding to chemotherapy (*p* < 0.05). Overexpression of *GLO1*, which encodes for glyoxalase 1, is closely associated with the occurrence of multidrug resistance in the context of not only tumorigenesis (39) but also infections caused by microorganisms (40). It plays a critical role in the development of innate and acquired drug resistance, and in cancer, overexpression of the *GLO1* gene and protein is characteristic of cells with high glycolytic rates (39). Thus, considering the enzymatic activity of glyoxalase 1 catalyzing the conversion of methylglyoxal, a natural antibiotic to glutathione D-lactate, it should be assumed that loss of response to treatment is associated with accumulation of glutathione D-lactate and reduction in glutathione (glyoxalase II) in the cells. This is accompanied by a decrease in the concentration of the substrate methylglyoxal, a cytotoxic byproduct of glycolysis that activates cell apoptosis (41). Sakamoto et al. confirmed significantly higher expression of GLO1 in human monocytic leukemia cell lines, i.e., UK711, K562/ADM, and UK110 cells. These authors indicated that GLO1 inhibits apoptosis of cancer cells by inactivating caspases treated with anticancer drugs, while noting that this may be a reversible effect (42). Interesting in this regard the study of Tamori et al., conducted on a breast cancer model, also confirmed that GLO1 expression is dependent on the histopathological grade of tumor malignancy (χ^2^ test, p = 0.002) and was significantly higher in basal cell breast cancer (43). Additionally, our study observed higher *GLO1* expression among patients with drug resistance, which is consistent with the observations of Alhujaly et al. who found that overexpression of this gene and protein reduces the antitumor properties of cisplatin, among others (44).

Moreover, we observed an increase in the mRNA and protein expression of TUFT1 in group A as compared with the control group and group B. However, we did not confirm that changes in the TUFT1 expression profile depend on the occurrence of drug resistance to platinum compounds in ovarian cancer. Thus, we speculate that TUFT1 may serve as a complementary molecular marker in differentiating the clinical stage of ovarian cancer. Such a conclusion seems reasonable taking into account observations made by Yang et al. who found that overexpression of TUFT1 mRNA and protein is characteristic for identifying higher clinical stages of colorectal cancer (stages III and IV) and development of vincristine resistance through the PI3K/AKT pathway (45). Dou et al. confirmed the association between TUFT1 overexpression and unfavorable prognosis among patients with intrahepatic cancer, which is directly related to HIF1-α overexpression and induction of oxidative stress (46).

The last mRNA that was differentiated in the test samples from control samples was the *STATH* gene, for which we noted decreased expression in cancer samples. To the best of our knowledge, this is the first study to report STATH expression in cancer samples. Two studies that evaluated the utility of STATH1 determination in clinical samples have been published thus far. The first one, by Sakurada et al., demonstrated the potential ability to differentiate nasal from vaginal secretions in forensic examinations based on the presence of the STATH1 protein (present only in nasal secretions) (47). The second one, by Gilbert and Stayton, indicated the presence of STATH1 in salivary secretions, where it participates in enamel mineralization and is produced by the human body in natural and recombinant forms (48).

We complemented our analysis of drug resistance-associated mRNA transcriptome by determining the expression pattern of miRNAs that potentially regulate the expression of the selected transcripts and testing this effect by determining the concentration of proteins encoded by the selected genes.

The influence of the selected miRNAs regulating the expression of the indicated mRNAs seems as feasible because for all of them the target score was >80. Thus, considering the results of the predictive analysis of the interaction between mRNA and miRNA and expression at the protein level, it seems correct that miRNAs not only act as negative regulators of expression at the post-transcriptional level but also can enhance expression, resulting in protein overexpression (49–51).

The analysis indicated that hsa-miR-133a-3p and hsa-miR-133b are molecules that regulate *UBA2* expression. The study published by Ukey et al. showed that assessment of hsa-miR-133a-3p levels may be a useful marker of oral squamous cell carcinoma risk (52). Additionally, the observations of Chang et al. are interesting, also showed that overexpression of hsa-miR-133a-3p in the sciatic nerve in rats was associated with more severe pain when compared with control rats. These authors also pointed out to the possibility of using the mentioned miRNA as a promising therapeutic target (53). Asai et al. reported a significant silencing of miR-133a-3p and miR-133b expression in head and neck squamous cell carcinoma samples (54), highlighting that the literature data indicate that miR-133a-3p and miR-133b acted as tumor-suppressive miRNAs (55).

Another miRNA involved in regulating expression of the selected genes is hsa-miR-561-5p, whose decreased expression in ovarian cancer samples resulted in protein overexpression in groups A and B compared with those in the control group. Our observations are in contrast to those in the reports of Chen et also demonstrated elevated expression of hsa-miR-561-5p in the liver cancer tissue, indicating that, along with miR-137, miR-149-5p is closely associated with the metastatic potential of liver cancer cells and the formation of lung metastases (56). Xi et al. found silencing of miR-561-5p expression in pancreatic ductal adenocarcinoma cell samples. The silencing of its expression resulted in decreased tumor cell proliferative potential, migration, and invasion (57).

This finding is supported by the complex nature of the miRNAs involved, where one miRNA in some tumor types is described as a pro-tumorigenic factor in one tumor lesion and as a tumor growth suppressor in another.

This situation was reported for miR-29, which was silenced in lung cancer and overexpressed in breast cancer samples (58). This may be because the same miRNA participates in different signaling pathways, which translates into the regulation of biological processes. In addition, nearly 60% of all mRNAs are regulated by miRNAs (59).

We observed that overexpression of hsa-miR-137-3p and hsa-miR-580-3p was involved in the regulation of *STATH* expression, with the silencing of STATH protein expression most likely in group A as a result of the aforementioned mRNA-miRNA interaction, while a contradicting outcome was observed in group B when compared with control samples. Considering that STATH expression is different in both groups, it is possible that the expression of the gene itself and the protein encoded by it, as well as miRNAs regulating its expression, depends on the clinical stage of the ovarian cancer lesions. In the case of miR-137-3p and hsa-miR-580-3p, it can be assumed that their expression is tissue specific, as Ding et al. confirmed that the silencing of miR-137-3p expression in colorectal cancer samples (60) and Dong et al. reported reduced expression in non-small cell lung cancer samples (61).

The last miRNAs evaluated are hsa-miR-1233-3p and hsa-miR-2052, whose expression was significantly higher in ovarian cancer samples than in control samples. Overexpression of miR-1233-3p was found among patients with renal cell carcinoma and was considered an adverse prognostic marker. Dias et al. determined the expression of specific miRNAs by using the liquid biopsy technique, which allows the determination of the concentration of selected biomolecules in body fluids, including blood, serum, and lavage. It plays a role in diagnosis and monitoring of therapy (62). miR-2052 has been described in the context of severe acute respiratory syndrome coronavirus 2 infection (63).

Thus, the analysis of drug resistance-associated miRNAs performed in this study indicates that the role of these regulatory molecules in the context of ovarian cancer has been insufficiently described.

In the last stage of our analysis, we summarize the risk factors significantly influencing the occurrence of drug resistance among patients with ovarian cancer. The most important were stage (OR: 69; 95% CI 6.32-753.61), *GLO1* mRNA overexpression (OR: 5.95; 95% CI 1.15-30.51), and ascites (OR: 2; 95% CI 0.49-8.32). The most significant factors predisposing to the development of ovarian cancer include menopause (OR: 10.64; 95% CI 4.17-27.1), TUFT1 protein overexpression (OR: 9.76; 95% CI 0.21-458.99), UBA2 protein overexpression (OR: 5.69; 95% CI 2.95-10.97), and GLO1 protein overexpression (OR: 5.68; 95% CI 0.18-180.09). Thus, it seems that screening diagnosis of ovarian cancer should be supplemented by GLO1, TUFT1, and UBA2 determination and assessment of the risk of loss of adequate response to chemotherapy by *GLO1* mRNA expression pattern determination. Of particular interest to clinicians should be the occurrence of ascites in oncology patients, as it significantly increases the risk of drug resistance to cisplatin.

As recent events surrounding the coronavirus disease pandemic have shown, understanding molecular mechanisms is invariably important and the development and introduction of commercially available diagnostic tests can be simple, effective, and useful.

Our study has both strengths and weaknesses. The strengths include the use of modern techniques to assess changes in mRNA and miRNA transcriptome expression, as well as the association of the observed changes with the concentration of proteins encoded by the selected genes. Although the sample size of the study and control groups may seem relatively small, it should be kept in mind that this is a single-center study, and its duration is short. Therefore, the study, although important, should be extended in the future.

## Summary

The analysis showed the greatest association with drug resistance for the following mRNAs and miRNAs: *UBA2* and hsa-miR-133a-3p, and hsa-miR133b; *GLO1* and hsa-miR61-5p; *STATH* and hsa-miR-137-3p, and hsa-miR-580-3p, *TUFT1* and hsa-miR-1233-3p, and hsa-miR-2052. The importance of determination of the biochemical markers CA-125 and HE4 in the diagnosis of ovarian cancer should not be marginalized. Our study suggests supplementing the current diagnostic approach by determining the expression profile of GLO1, TUFT1, and UBA2 and assessing the risk of loss of adequate response to chemotherapy by determining the *GLO1* mRNA expression pattern. Of particular interest to clinicians should be the occurrence of ascites in female cancer patients, which is an unfavorable prognostic factor because it significantly increases the risk of cisplatin resistance. Finally, we confirmed the validity of molecular assessment and the fact that molecular changes precede phenotypic changes, as we determined changes in gene, miRNA, and protein expression in cancer samples before the finding of cisplatin drug resistance among ovarian cancer patients.

## Data availability statement

The datasets presented in this study can be found in online repositories. The names of the repository/repositories and accession number(s) can be found in the article/supplementary material.

## Ethics statement

The studies involving human participants were reviewed and approved by Bioethical Committee operating at the Regional Medical Chamber in Kraków, no. 185/KBL/OIL/2020 and 186/KBL/OIL/2020, dated September 20, 2020. Informed consent was obtained from all patients. The patients/participants provided their written informed consent to participate in this study.

## Author contributions

Conceptualization, MO and BG; methodology, MO and AŚ; software, EN; investigation, MO, BG, and AŚ; resources, DB; data curation, BG; writing-original draft preparation, PJ, MO, BG; writing-review and editing, RS; supervision, MO and BG; project administration, BG and MO. All authors have read and agreed to the published version of the manuscript.

## Acknowledgments

We would like to thank Nikola Zmarzły, PhD for helping with data analysis and improving our paper.

## Conflict of interest

The authors declare that the research was conducted in the absence of any commercial or financial relationships that could be construed as a potential conflict of interest.

## Publisher’s note

All claims expressed in this article are solely those of the authors and do not necessarily represent those of their affiliated organizations, or those of the publisher, the editors and the reviewers. Any product that may be evaluated in this article, or claim that may be made by its manufacturer, is not guaranteed or endorsed by the publisher.
